# Strategies for Improving Postpartum Contraception Compared With Routine Maternal Care: A Systematic Review and Meta-Analysis

**DOI:** 10.3389/ijph.2023.1605564

**Published:** 2023-04-13

**Authors:** Denghui Hu, Yuxiang Tang, Kaiyan Pei

**Affiliations:** ^1^ National Research Institute for Family Planning, Chinese Academy of Medical Sciences, Beijing, China; ^2^ School of Population Medicine and Public Health, Chinese Academy of Medical Sciences & Peking Union Medical College, Beijing, China

**Keywords:** systematic review, family planning, contraception, postpartum, maternal care, meta-analysis

## Abstract

**Objectives:** This study aimed to systematically review the effectiveness of service interventions for improving postpartum contraception, including contraceptive use, prevention of repeat pregnancies and induced abortions.

**Methods:** A systematic literature search was conducted in three databases until June 2022 (PROSPERO registration CRD42022328349). Estimates of intervention effects from meta-analyses were represented as odds ratios (OR) with 95% confidence intervals (CI).

**Results:** 16 studies with 14,289 participants were included, with four kinds of interventions recognized. Interventions effect in increasing use of contraceptives and decreasing rates of repeated pregnancy for up to 6 months postpartum (OR = 2.24, 0.06, 95% CI = 1.46–3.44, 0.02–0.22, respectively), with no significant associations with contraceptive use at 12 months postpartum, prevention of postpartum repeat pregnancies and induced abortions during 1 year after childbirth.

**Conclusion:** We concluded that interventions impact the initiation of postpartum contraceptive use and prevention of repeat pregnancy with an overall certainty from low to moderate. These findings highlight the need for additional studies to integrate the beneficial effect of several interventions and then design more feasible strategies, which is important for the maternal and child healthcare systems.

## Introduction

Although a rapid increase in the effectiveness of contraceptives has been achieved worldwide, the rates of perfect or typical use have not shown similar improvements. Moreover, the percentage of pregnancies considered to be unintended remains substantially higher in some regions (1, 2). According to a study that evaluated data from nationally published studies and official statistics, there were an average of approximately 121.0 million unintended pregnancies each year globally, which translates to a rate of 64 unintended pregnancies per 1,000 women aged 15–49 years in 2015–19 (3, 4). Studies also revealed that many unintended pregnancies are terminated, with more than half of the procedures performed unsafely (3, 5, 6).

Postpartum women are among those with the higher risk of unintended and closely spaced pregnancies and the greatest need for limiting birth. The extended postpartum period is the first year after childbirth (7). Maternal healthcare, through family planning services and *via* modern contraceptive methods, can allow postpartum women to prevent unintended pregnancies and determine their pregnancies’ spacing during this period (8–10). This kind of prevention of unintended pregnancies also helps to lower maternal ill-health and the number of pregnancy-related deaths (11). Previous studies (12, 13) suggested that at least 30% of maternal deaths and 10% of neonatal deaths can be avoided when the next pregnancies of mothers are delayed for 2 years after delivery. Given that, abundant interventions and strategies of services were applied internationally as vital components of comprehensive maternal healthcare to available postpartum family planning (PPFP) uptake.

Relevant interventions include counselling, education, reminding massages, etc., which may be provided to individuals or groups in a hospital/clinic or during home visits at prenatal care or postnatal care. The counselling strategies consist of a single component or multiple components delivered in a single session or multiple sessions at various contact points (including the prenatal care clinic, during delivery, postnatal care, etc.). The educational interventions used to be provided through oral/written materials, mobile phones, or multiple media to disseminate maternal care and family planning information in person or through a group discussion. The reminder messages may be sent daily to remind women to take their pills or may be sent reminders when their next injection or medical visit is due. Although these interventions were internationally implemented, the Royal College of Obstetricians & Gynecologists (RCOG) still believes that PPFP is often ignored, and many biases and misconceptions have limited its availability (14).

Several non-randomized trials (NRs) have shown that interventions delivered in maternal care settings may improve adherence to contraceptive use and reduce repeat pregnancy and induced abortion during the postpartum period (15–17). Furthermore, a systematic review assessed the impact of educational interventions based on these NRs, which concluded that education might increase postpartum contraceptive use. However, the quality of evidence was moderate to low, and interventions must be improved by strengthening the program design and implementation (18). Despite existing research, there is a lack of randomized controlled trials (RCTs), and systematic reviews assessing the evidence from RCTs are limited (4, 19, 20). And more is known about the appropriateness of specific contraceptive methods for postpartum women than how to help women use certain contraceptives. To address this gap, the current systematic review was conducted to reach consensus opinions about the impact of service interventions from RCTs and guide future research and clinical practices on the appropriate strategies for improving postpartum contraceptive use to make future maternal healthcare more valid and reliable.

## Methods

This study followed the Preferred Reporting Items for Systematic Reviews and Meta-Analyses (PRISMA) guidelines (21), and its protocol was registered in the International Prospective Register of Systematic Reviews (PROSPERO) database: CRD42022328349.

### Eligibility Criteria

#### Population

For the present review, studies that recruited postpartum women 18 years or older without being infected with human immunodeficiency virus (HIV) and another special pregnant status were eligible for inclusion.

#### Intervention

Service interventions scoping to improve postpartum contraception were included. For the purpose of this review, the intervention could start at any point in maternal healthcare, including prenatal care, postnatal care, or child healthcare.

#### Comparisons

Any comparison group without specific PPFP interventions was acceptable, including local routine maternal care or no interventions.

#### Outcomes

The primary outcome was rates of contraceptive use among postpartum women during 1 year after childbirth, compared to control groups. The secondary outcomes were the rates of repeat pregnancies and induced abortions during the postpartum period.

#### Study Design

Only randomized controlled trials and cluster-randomized trials in full-text published in English were eligible for inclusion. Studies whose follow-up time was less than 6 months after delivery; master’s or doctoral thesis or research report; studies that did not report health-related outcomes; multiple submissions and duplicate publications were excluded.

#### Search Strategy

English electronic databases (PubMed, EMBASE, and Web of Science) were comprehensively and systematically searched from their inception until June 2022. In-progress trials were searched for on the clinical trials register. Finally, bibliographies of the retrieved articles were also hand-searched to identify any relevant articles for our review. The search terms used were reported in Supplementary Tables S1–S3.

#### Data Screening and Extraction Process

Two authors (DH and YT) were assigned to independently screen the titles and abstracts among the records organized in Endnote X9 to retrieve relevant records. Then, they were also assigned to independently perform the second screening of the full text based on the predefined inclusion criteria, and independently extracted data from included studies using a format prepared in a Microsoft Excel spreadsheet. Detailed information was extracted, including author, publication year, location, study design, sample size, intervention details, comparison condition, service opportunities, and outcomes. Discrepancies and disagreements during the entire process were resolved by the third author (KP). Eligibility for inclusion in the meta-analysis was also determined for each study.

#### Risk of Bias

We evaluated the quality of the studies based on the Cochrane “risk of bias” assessment tool and using criteria outlined in the Cochrane Handbook for Systematic Reviews of Interventions (22). Based on the rating obtained from 5 domains, each study was classified as having “Low risk,” “High risk,” and “Some Concerns.” The risk of bias was assessed by two authors (DH and KP) independently. Any discrepancies were discussed until a consensus was reached. A summary figure of the assessed bias of the included studies was created using Microsoft Excel (Microsoft Corp, Redmond, WA, USA).

#### Publication Bias and Heterogeneity

Rigorous searches (electronic/database search and manual search) have been used to minimize the risk of bias. With recommendations for examining and interpreting funnel plot asymmetry in meta-analyses of randomized controlled trials (23), we could not construct funnel plots to test for publication bias as there were insufficient studies for any one comparison. According to the Cochrane handbook criteria, the Higgins I^2^ test measured heterogeneity among studies with its corresponding *p*-value. I^2^ test statistics values of 0, 25, 50, and 75% were considered no, low, moderate, and high degrees of heterogeneity, respectively (24). In this study, when I^2^ > 50%, there is an obvious heterogeneity and the random effect model will be used, otherwise, the fixed effect model will be applied.

#### Data Synthesis

Rates of postpartum contraceptive use, repeat pregnancy, and induced abortion during 1 year after childbirth were expressed as odds ratio (OR) and 95% confidence interval (CI). Where studies measured the same outcomes (including rates of contraceptive use, rates of repeat pregnancy, and rates of induced abortion during the postpartum period), we included them in a meta-analysis. Three random-effects models were applied to pool the ORs of interventions on contraceptive use at 6/8/12 months postpartum compared with local routine care or no interventions. Two fixed-effects models were applied to calculate the pooled OR for impact on repeat pregnancy. The results were presented using texts and forest plots. Sensitivity analysis was conducted using leave-one-out influence analyses with a *p*-value of less than 0.05 declaring the statistical significance. The data syntheses were done using R-studio Version 1.1.383 (1999 Free Software Foundation, Boston, Massachusetts, MA, USA: RStudio, PBC).

## Results

### Literature Search

**FIGURE 1:**
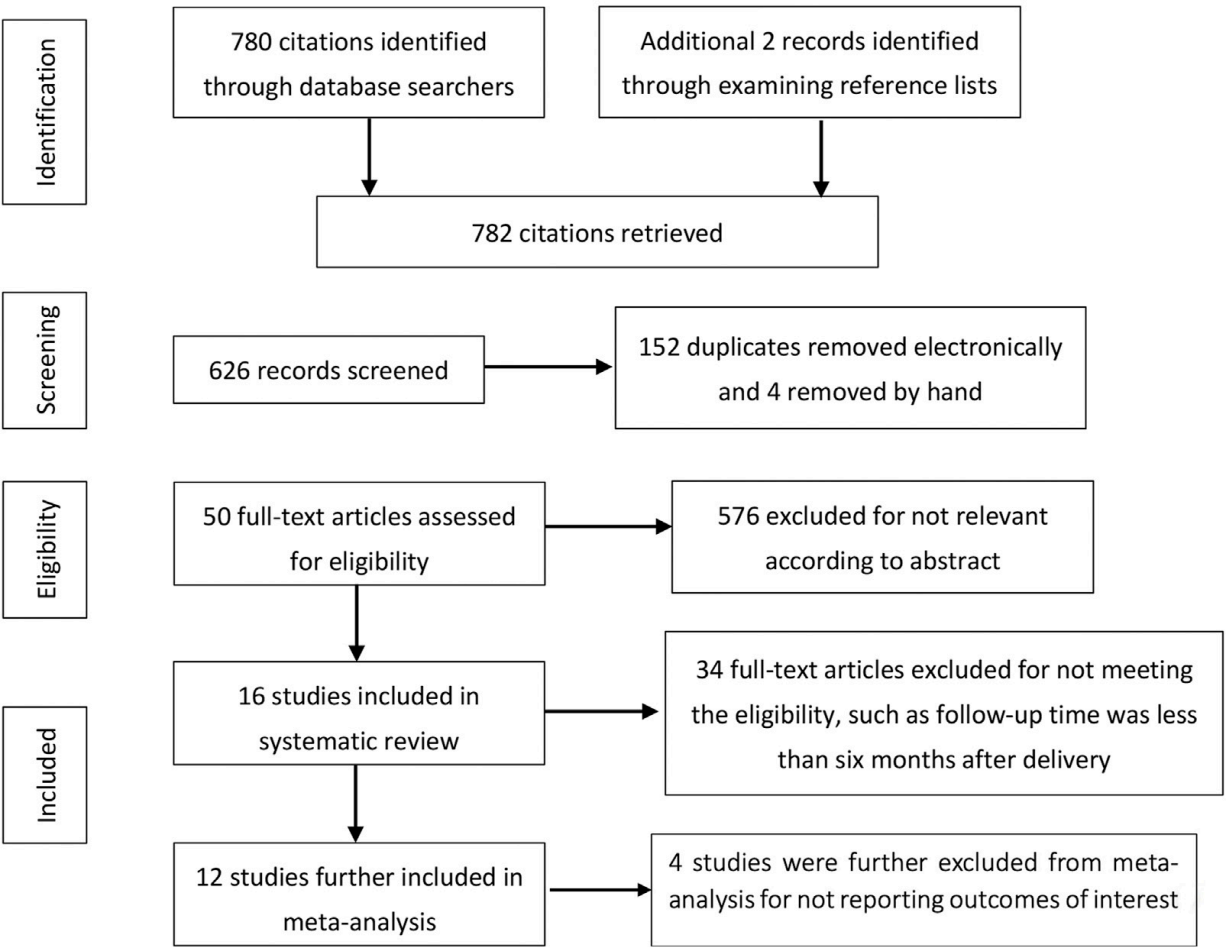
Flowchart of literature search (Beijing, China. 2022). Notes: The flow diagram was based on PRISMA.

782 citations were identified through database searches and reference lists of relevant articles. 152 duplicates were removed electronically, and we removed 4 by hand, leaving 626 unduplicated citations. After discarding 576 for not meeting the eligibility criteria according to titles and abstracts, we reviewed the full text of 50 articles for eligibility. Then, we excluded 34 and included 16 articles. 12 of 16 studies reported the outcomes of interest were included in the meta-analysis, and 4 eligible articles, excluded from the meta-analysis since they did not report any outcome of interest but other health-related outcomes, were included as part of the systematic review. A PRISMA flowchart was reported in Figure 1.

#### Characteristics of Included Studies

The characteristics of included studies are summarized in Table 1. Of the 16 studies, 12 were randomized controlled trials, and 4 were cluster-randomized trials. A total of 4 kinds of intervention strategies were included. Ten studies, including 8,463 women, provided direct in-person counselling, including one-to-one counselling, counselling sessions, and specific counselling meeting. Five trials provided written educational materials or videos and included 3,137 women. Five (N = 3,382) provided intensive reminders of appointments or dosing through sending a short message, phone call, or self-developed system. Studies also provided other healthcare services such as home visits, health screening tools, and free contraceptives.

**TABLE 1:** Main characteristics of the included studies (Beijing, China. 2022).

Study	Location	Study design	Sample size		Intervention^*^	Comparison	Opportunity	Outcomes^*^
			T	C				
(25)	Nepal	RCT	135/135/135	135	B	F	Prenatal care	a, e
(26)	Scotland/China/South Africa	RCT	171/254/192	214/263/197	A	E	Prenatal care	b, c, d, e
(27)	USA	RCT	182/141	67/78	B, D	E	Postnatal care	c
(28)	China	RCT	998	1,002	D	F	Postnatal care	b, c
(29)	Turkey	RCT	50	97	A	B	Prenatal care	e
(30)	Nigeria	RCT	108	108	A	E	Prenatal care	a, e
(31)	Egypt	RCT	579	579	A, D	A	Postnatal care	a, e
(32)	Uganda	RCT	627	758	A, D	E	Prenatal care	e
(33)	Burkina Faso	RCT	583	561	A, B	E	Prenatal + Postnatal care	e
(34)	Burkina Faso	CRT	286	285	A, C	E	Prenatal + Postnatal care	a, b, e
(35)	Kenya	RCT	130	130	C, E	E	Postnatal care	a, e
(36)	USA	CRT	197	218	B, D	B	Child healthcare	a, b, c, e
(37)	Spain	CRT	493	482	A, B, C, E	E	Prenatal + Postnatal care	a, b, e
(38)	Congo	CRT	286	290	A, C	E	Postnatal care	b, e
(39)	Egypt	RCT	500	500	A, C	A	Postnatal care	a, e
(40)	Malawi	RCT	1026	1117	A	E	Prenatal + Postnatal care	e

NOTE: RCT, randomized controlled trial; CRT, cluster-randomized trial. *Interventions: A, Counseling (including one-to-one counseling, extra counseling, specific counseling, etc.); B, Education (including educational materials, video, leaflet, booklet, discussion meeting, etc.); C, Reminder (including short message service, reminder, invitation, etc.); D, Other visit care (including a home visit, health screening tools, provision of contraceptives, etc.); E, Routine care (local routine care); F, without any intervention. *Outcomes: a, contraceptive use at 6 months postpartum; b, contraceptive use at 12 months postpartum; c, rate of repeat pregnancy during 1 year after childbirth; d, rate of induced abortion during 1 year after childbirth; e, other health-related outcomes postpartum.

Of these eligible studies, ten compared a compound strategy with the local routine healthcare of impact on the adoption of postpartum contraceptives. In contrast, others (*n* = 6) only provided a simple intervention such as counselling, educational materials, or screening tools, respectively. Only one study provided educational interventions and health screening tools for service opportunities in child healthcare clinics. Others (including 15 articles) were conducted in prenatal clinics, postnatal clinics, or both.

#### Risk of Bias Assessment

**FIGURE 2:**
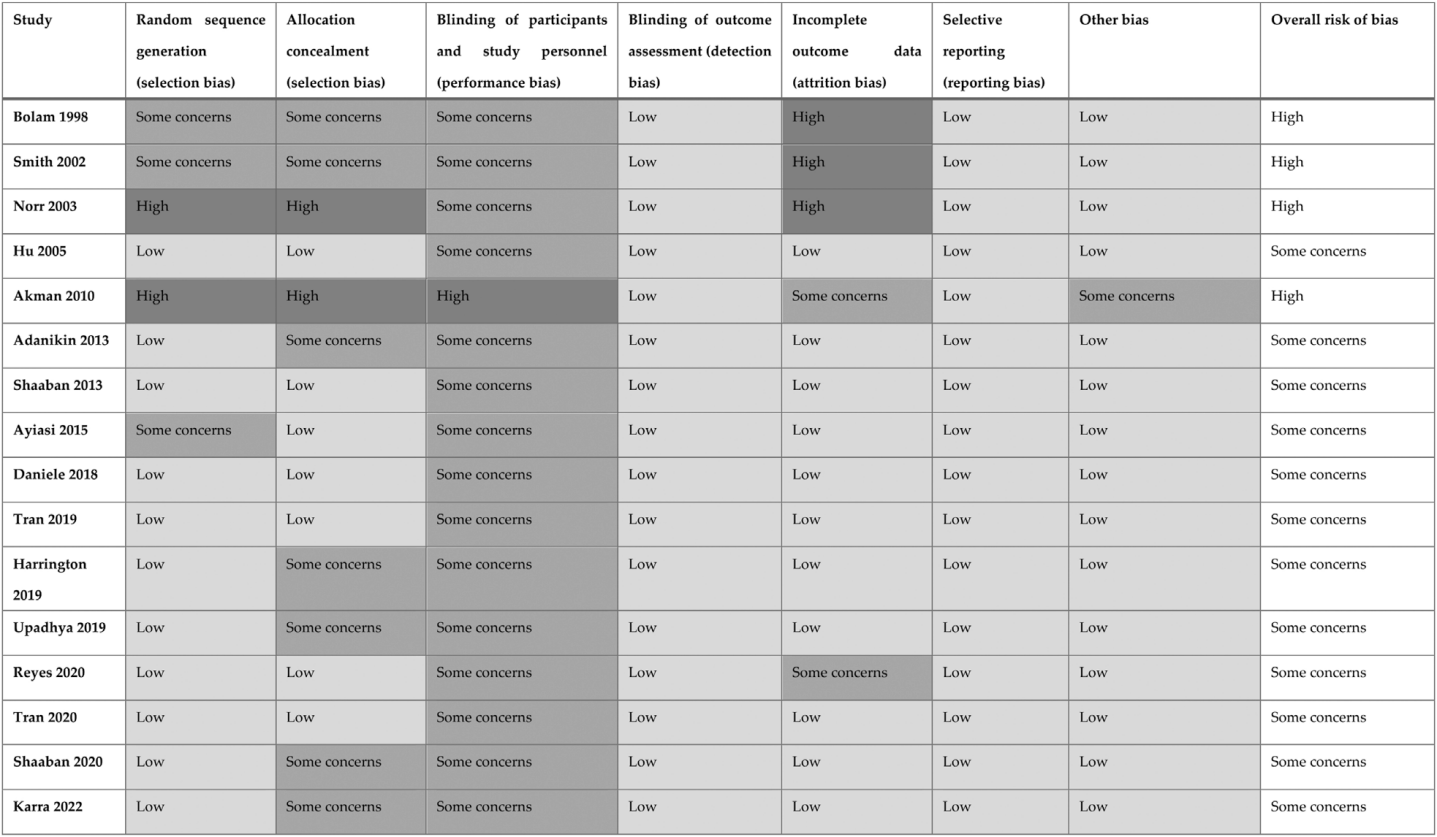
Risk of bias ratings (Beijing, China. 2022). Notes: low, low risk of bias; high, high risk of bias.

We found that 5 out of 16 studies had high risk or some concerns over the randomization process, all studies had high risk or some concerns over the blinding process, all studies had low risk over the outcome assessment and 5 studies had a high risk or some concerns over the missing of outcome data. Only 1 study had some concerns over other potential biases. Overall, 4 out of 16 studies had a high risk of bias and the other 12 studies had some concerns, and the quality of evidence was moderate to low (Figure 2).

### Outcomes

#### Contraceptive Use

**FIGURE 3:**
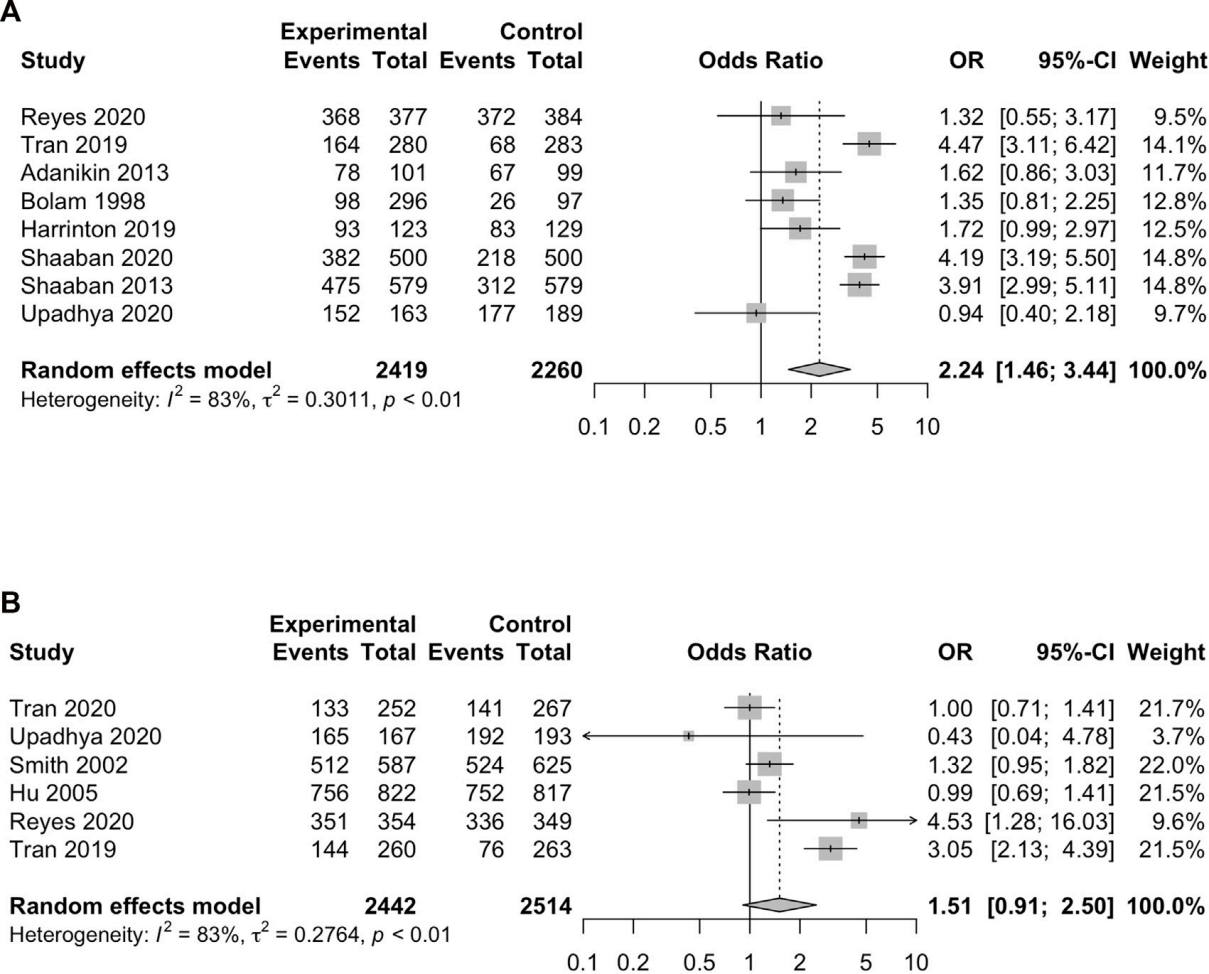
Forest plots of rates of contraceptive use **(A)** at 6 months postpartum, **(B)** at 12 months postpartum (Beijing, China. 2022). Notes: The squares depict individual study point estimates of OR. Horizontal lines display the 95% CI for point estimates.

Eight studies examined the impact of interventions on contraceptive use at 6 months postpartum compared with routine healthcare or no intervention. Three showed a statistically significant increase in contraceptive uptake among those who revived services. The remaining did not show a significant difference in contraceptive use. In the meta-analysis, it appears that interventions are significantly associated with increased rates of contraceptive use at 6 months postpartum (Figure 3A; OR = 2.24, 95% CI = 1.46–3.44, I^2^ = 83%, N = 4,679), based on the random effect model. Leave-one-out influence analyses indicated that the findings of the meta-analysis did not rely on a particular study.

Six studies (N = 4,956) compared interventions versus routine healthcare on contraceptive use at 12 months postpartum. Based on the random effect model (I^2^ = 83%, *p* < 0.01), the pooled results gave an OR of 1.51, favouring more chosen contraceptives. However, the 95% CIs were 0.91–2.50, indicating no significant influence of interventions on postpartum contraceptive uptake in Figure 3B. Leave-one-out influence analyses indicated that the findings of the meta-analysis did not rely on a particular study.

In addition, Hu and Daniele (28, 33) reported conflicting findings on contraceptive use at 8 months postpartum. After combining these two studies, the meta-analysis showed no significant associations with interventions (OR = 0.97, 95% CI = 0.52–1.81, I^2^ = 91%, N = 2,919) based on a random effect model. Karra et al. (40) found that contraceptive use after 2 years of intervention exposure increased by 5.9 percentage points compared with the control group. Of the two remaining articles that reported postpartum contraception, neither showed a statistically significant difference in interventions associated with any contraceptive uptake at 6–9 months or 12–14 months postpartum.

#### Repeat Pregnancy

**FIGURE 4:**
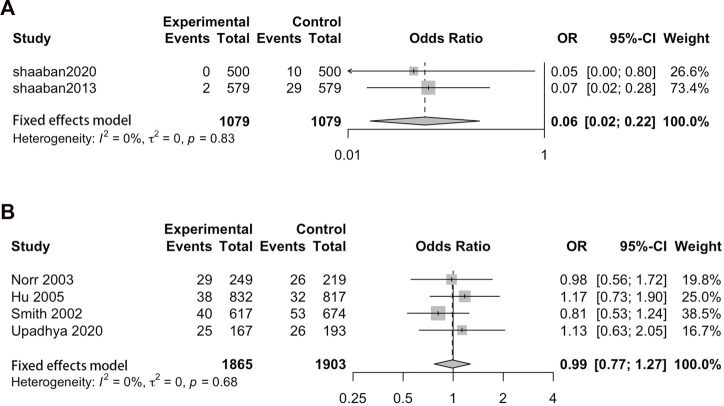
Forest plots of the rate of repeat pregnancy **(A)** during 6 months postpartum, **(B)** 1 year after delivery (Beijing, China. 2022). Notes: The squares depict individual study point estimates of OR. Horizontal lines display the 95% CI for point estimates.

Two articles evaluating the repeat pregnancy rates during 6 months postpartum reported a significant association. The meta-analysis also showed a statistically significant decrease in repeat pregnancy (Figure 4A. OR = 0.06, 95% CI = 0.02–0.22, I^2^ = 0%, N = 2,158) after receiving services. Four articles evaluated repeat pregnancy or unintended pregnancy 1 year after childbirth. However, none illustrated a statistically significant difference between the intervention groups and routine care or no intervention groups in Figure 4. Based on a fixed-effect model, the results also showed no statistically significant association between repeat pregnancy and interventions (Figure 4B. OR = 0.99, 95% CI = 0.79–1.24, I^2^ = 0%, N = 3,768). Leave-one-out influence analyses indicated that the findings of the meta-analysis did not rely on a particular study. However, one study in Malawi (40) reported the intervention group’s hazard of repeat pregnancy was significantly lower during 24 months postpartum.

#### Induced Abortion

Only one study reported the rate of induced abortion 1 year after delivery. The study was simultaneously conducted in Edinburgh, Cape town, and Shanghai. Only 2 participants and 1 control in Edinburgh and 29 participants and 27 controls in Shanghai experienced induced abortion. And the results showed no significant influence of interventions on rates of induced abortion during 12 months after childbirth.

## Discussion

This systematic review summarizes the strategies adopted by previous RCTs, provides a synthesis of family planning services on the impact on postpartum contraception and finds that implementing interventions significantly impacts the use of contraceptives and prevention of repeat pregnancy for up to 6 months postpartum compared with local routine healthcare. These findings may offer significant recommendations for improving postpartum care and childcare systems.

Despite variations in strategies, half of the studies that reported rates of contraceptive use indicated an improved initiation of contraception, especially modern contraceptive uptake. Family planning services delivered *via* counselling (one-to-one, session and et al.), education, reminder and health visit during the prenatal and postpartum period were associated with increased contraceptive use. The meta-analysis shows a significantly positive association with contraceptive use at 6 months after childbirth. In addition, two studies reported a significant decrease in the repeat pregnancy rate 6 months after delivery as well as the result of the meta-analysis indicates a significantly negative association.

Although 2 of 6 studies reported a significantly increasing association with contraceptive use at 12 months postpartum, the meta-analysis shows no significant associations. The results of the prevention of repeat pregnancy and induced abortions 1 year after childbirth were also insignificant, regardless of the individual or meta-analysis.

Therefore, there are observed associations that indicated family planning services improve the initiation of postpartum contraception, without impacting on prevention of repeat pregnancy and induced abortion 1 year after childbirth. But there is a caveat that a certain degree of heterogeneity among the studies, and due to the limitation of the number of studies included, we have not well distinguished the causes of heterogeneity and impossible to conduct subgroup analysis. Another warning is that these estimates are inaccurate for these studies, with about half, being conducted with small samples (n<200). Thus, more studies with an adequate sample on the effects of family planning services on postpartum contraceptive uptake among postpartum women are needed.

Whether the goals are short-term or long-term will vary according to population differences, for example, the women’s age and number of children. Similarly, differences in the design among studies will also affect the goals. But included studies did not always consider the age differences and provide essential information, such as the frequency, theories and other details of interventions. Therefore, it may be unreasonable to evaluate the long-term effect (such as prevention of repeat pregnancies and induced abortions during 12 months postpartum) of intervention based on existing evidence and more clarity is also needed for future studies to consider the difference in the study design, intervention details and population.

In addition, RCOG believes family planning should be discussed from the beginning of pregnancy until the end of the postpartum. The current study includes three opportunities including prenatal care, postnatal care and child healthcare. However, there was also a lack of studies conducted during delivery and in child healthcare clinics, for which we were unable to obtain more evidence on the impact of the different contact points. Hence, we recommend more rigorous studies are needed to explore the feasibility and effectiveness of postpartum family planning services at different opportunities, and also an integrated delivery system or strategy of maternal healthcare, family planning services, and child healthcare during the whole postpartum period is needed to provide evidence-based guidance for future clinical postpartum contraceptive services and maternal and child healthcare practices.

Overall, this systematic review comprehensively synthesizes the available evidence, mainly on family planning services’ effects on postpartum contraception. This systematic review strengthens the recommendations that women receive family planning services integrated with routine maternal health services in prenatal care clinics, postnatal clinics and child healthcare clinics. In addition, this review will have significant implications for designing strategies to enhance family planning services to improve contraceptive uptake and maternal and child health.

### Limitations

Several limitations of our systematic review should be highlighted. First, the primary studies included in this review were limited to some regions of the country, and others might be underrepresented. Second, only articles published in English were considered to conduct this review, which may result in missing studies that could have been published in other languages. Meanwhile, another strong limitation of the meta-analysis is that only 3 academic search systems were used, which greatly limited the application of evidence. And then, we did not distinguish interventions between different kinds under the same category, which makes we were not entirely certain which intervention or interventions were responsible for the significant differences observed in the meta-analysis. Few primary studies with a small sample size were included and we did not include grey literature in the review, which might influence the estimated magnitude of postpartum modern contraceptive use. Because the limited number of included studies leads the further subgroup analysis not being possible in each group. In addition, differing time frames likely influenced the design, intervention content, and outcome measures.

### Conclusion

Counselling, education, reminders, and other related interventions in prenatal care clinics, postnatal care clinics, or both were significantly associated with improving postpartum contraception for up to 6 months, although the certainty of evidence was low to moderate. Considering the uptake of postpartum contraceptive methods is critical to reducing maternal and neonatal mortality and morbidity. It should be promoted and strengthened that family planning services to all women in prenatal care, during delivery, postnatal care, child healthcare or all. Furthermore, further investigation of detailed family planning services’ effects in different contact points on contraception among women within the 12 months postpartum period is needed.
