# Supplementary material for: Strategies for Improving Postpartum Contraception Compared With Routine Maternal Care: A Systematic Review and Meta-Analysis
Source: Int J Public Health. 2023 Apr 13;68:1605564. doi: 10.3389/ijph.2023.1605564 (PMC10133502; doi:10.3389/ijph.2023.1605564)
Supplement: Supplementary file 1 [file Table1.PDF]

Table S1. Search strategy (for PubMed).

Search terms	Counts
e.g., For PubMed (1970 Low Date of search)	
1. “Contraception” [Mesh]	
2. “Contraception Behavior” [Mesh]	
3. “Contraceptive Agents” [Mesh]	
4. “Contraceptive Devices” [Mesh]	
5. “family planning services”	
6. [#1 or #2 or #3 or #4 or #5]	100387
7. educat*	
8. counsel*	
9. communicat*	
10. “information dissemination”	
11. intervention*	
12. choice	
13. choose	
14. use	
15. [#7 or #8 or #9 or #10 or #11 or #12 or #13 or #14]	8759341
16. “Postpartum Period” [Mesh]	
17. “Postnatal Care” [Mesh]	
18. postpartum	
19. post-partum	
20. postnatal	
21. “repeat pregnancy” [tiab]	
22. mothers [ti]	
23. [#16 or #17 or #18 or #19 or #20 or #21 or #22]	274978
24. [#6 and #15 and #23]	
25. article type = clinical trial or randomized controlled trial	189

Table S 2. Search strategy (for Embase).

Search terms	Counts
("Contraception" OR "Contraception Behavior" OR "Contraceptive Agents" OR "Contraceptive Devices" OR "family planning") AND ("educat*" OR "counsel*" OR "communicat*" OR "information dissemination" OR "intervention*" OR "choice" OR "choose" OR "use") AND ("Postpartum Period" OR "Postnatal Care" OR "Postpartum" OR "repeat pregnancy")	
Restrict to clinical trial, randomized controlled trial	414

Table S 3. Search strategy (for Web of Science).

Search terms	Counts
TS= (("Contraception" OR "Contraception Behavior" OR "Contraceptive Agents" OR "Contraceptive Devices" OR "family planning") AND ("educat*" OR "counsel*" OR "communicat*" OR "information dissemination" OR "intervention*" OR "choice" OR "choose" OR "use") AND ("Postpartum Period" OR "Postnatal Care" OR "Postpartum" OR "repeat pregnancy"))	
Restrict to clinical trial	177
